# Otolaryngology exposure in a longitudinal integrated clerkship setting

**DOI:** 10.1186/s40463-017-0215-1

**Published:** 2017-07-10

**Authors:** Grace Margaret Scott, Corliss Ann Elizabeth Best, Damian Christopher Micomonaco

**Affiliations:** 1 Laurentian University, 935 Ramsey Lake Rd, Sudbury, ON P3E 2C6 Canada; 2 Northern Ontario School of Medicine, 935 Ramsey Lake Rd, Sudbury, ON P3E 2C6 Canada

**Keywords:** Undergraduate medical education, Curriculum development, Longitudinal integrated clerkship, Primary care, Otolaryngology

## Abstract

**Background:**

Although 20–40% of primary care complaints are Otolaryngology-Head and Neck Surgery (OtoHNS) related, little emphasis is placed on OtoHNS instruction at the undergraduate medical education level. An OtoHNS clerkship rotation is not required at most Canadian medical schools. Furthermore, at institutions offering an OtoHNS rotation, less than 20% of students are able to complete a placement. Given that a large percentage of medical students in Canada will pursue primary care as a career, there remains a gap in providing OtoHNS clinical training. During the longitudinal integrated clerkship at the Northern Ontario School of Medicine (NOSM), students are assigned to one of 14 sites, and not all have access to an otolaryngologist. This study looks to quantify the level of exposure students are receiving in OtoHNS at NOSM and to assess their comfort level with diagnosing and treating common otolaryngologic conditions.

**Methods:**

A structured 13-item survey was administered to second, third and fourth year medical students at NOSM.

**Results:**

A majority (67.9%) of medical students surveyed had not observed an otolaryngologist. Furthermore, most students (90.6%) reported receiving very little OtoHNS classroom based and clinical instruction during medical school.

**Conclusions:**

A discrepancy exists between the quantity and breadth of OtoHNS training received in undergraduate medical education and the volume of OtoHNS encounters in primary care practice. Although geographic dissemination of students in the distributed learning model may be a challenge, strategies such as standardized objectives and supplemental electronic resources may serve to solidify clinical knowledge.

**Electronic supplementary material:**

The online version of this article (doi:10.1186/s40463-017-0215-1) contains supplementary material, which is available to authorized users.

## Background

Problems related to the ear, nose and throat are frequently encountered across a variety of medical disciplines. In primary care, these conditions represent up to 25% of the practice workload [1]. Notwithstanding the quantity of otolaryngologic concerns, it is generally underrepresented in undergraduate medical education (UME) [2–5]. Furthermore, a recent review found that the majority of final year medical students and junior doctors in the United Kingdom are not confident in managing patients with common Otolaryngology-Head and Neck Surgery (OtoHNS) problems [6].

In Canada, the OtoHNS curriculum is not standardized across medical schools [5, 7]. There is currently great variability in the organization of teaching blocks and clerkship opportunities [5], such that the majority of Canadian medical students are never exposed to a clinical rotation in OtoHNS as part of their training [7]. Adequate exposure may present an increased challenge in a longitudinal integrated clerkship (LIC) model where not all 14 sites have access to an otolaryngologist. The Northern Ontario School of Medicine (NOSM) in Canada is the first medical school in which all students undertake LIC clinical training within a programme known as the Comprehensive Community Clerkship (CCC) [8, 9]. The Northern Ontario School of Medicine remains the only Canadian medical school that requires all students to complete their third year clinical training this way. Beyond specialist availability, students are also limited in their OtoHNS exposure due to the limited time allotted for specialty placements in this curriculum model.

Longitudinal integrated clerkships afford students the opportunity to develop meaningful therapeutic relationships with patients and trusting, collegial relationships with their preceptors [10–14]. Graduates of LIC clinical training have been shown to perform as well as their peers in traditional rotation-based programs [13, 15]. Advantages of a LIC include improved academic results, [16–18] enhanced patient-centredness, [16, 17] and more meaningful learning relationships [8, 19]. Compared to traditional rotation based clerkships in tertiary hospital settings, medical students in a LIC have increased exposure to core clinical conditions [20].

This study looks to quantify the level of OtoHNS exposure students are receiving in a LIC model, and to assess their comfort level with diagnosing and treating common otolaryngologic conditions.

## Methods

Ethics approval was obtained from both Laurentian University and Lakehead University for this cross-sectional survey design study.

### Subjects

Inclusion criteria were undergraduate medical students at NOSM currently enrolled in years 2–4 of the program.

### Survey design

We developed a questionnaire modeled on a previous study with family medicine [21]. This questionnaire was developed for resident learners to include twenty-one OtoHNS related issues, and seventeen OtoHNS procedural skills. Though all clinical presentations and procedural skills were included in the study’s questionnaire, a priori decisions were made by the authors to focus on those clinical presentations and procedural skills that would be most appropriate for undergraduate medical learners to understand. These focused items included the following clinical presentations: otitis media, rhinitis and sinusitis, hoarseness, dysphagia, gastroesophageal reflux, thyroid nodules, salivary gland disease, peritonsillar abscess, sensorineural hearing loss, recurrent otitis media, and sleep apnea/snoring. Of the procedural skills, the following were highlighted as being appropriate for the undergraduate medical learner: otoneurologic exam, anterior rhinoscopy/nasal exam, oral cavity exam, indirect laryngoscopy, interpretation of an audiogram, explanation of common procedures and finally fine needle aspiration biopsy. The comprehensive survey asked medical students about: (1) their exposure to OtoHNS, (2) amount of training to date, and (3) perceived knowledge and ability to manage OtoHNS related clinical presentations. Students were also asked to identify their year of study and clinical clerkship site if they were in their clinical year of study. The survey was expected to take ten minutes to complete. A complete copy of the questionnaire can be found in Additional file 1: Appendix 1.

### Survey distribution

In March 2016, an invitation to participate and link to the survey was electronically sent to all year 2–4 NOSM students by the academic class representatives. The electronic link led the students to a Survey Monkey questionnaire. The email invitation included an attached letter of information, as did the first page of the electronic survey. Survey responses were voluntary and anonymous. In order to optimize the response rate, a modified Dillman approach [22] was used. Specifically, an email reminder containing the survey link was sent to all students two-weeks and four-weeks following the initial invitation.

### Data analysis

Descriptive statistics were generated using Microsoft *Excel*.

## Results

**Fig. 1:**
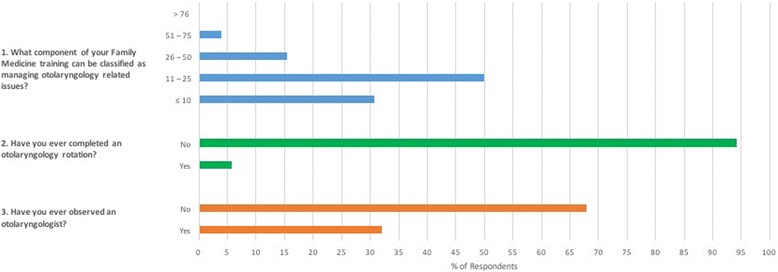
Have you ever observed an otolaryngologist? Have you ever completed an otolaryngology rotation? What component of your Family Medicine exposure can be classified as managing Otolaryngology associated issues?

In total, 53 participants completed the online survey. This afforded a response rate of 27.6% (53/192 enrolled students). Representation was gained from the three years of study, as well as all 14 CCC sites (Table 1). This included 23 (43.4%) second year students, 14 (26.4%) third year students and 16 (30.2%) fourth year students. The survey did not contain a question on gender. The majority of students that responded (67.9%) had not observed an otolaryngologist and an even greater proportion (94.2%) had not completed an OtoHNS rotation (Fig. 1). In addition, 69.2% of students felt that a significant portion (≥ 11%) of their family medicine exposure included OtoHNS associated presentations (Fig. 1).

**Table 1:** Participant characteristics

Item	Participants
Year of study (*n*,% total)	
2	23 (43.4)
3	14 (26.4)
4	16 (30.2)
CCC Site (*n*,% senior students)	
Bracebridge	1 (3.3)
Dryden^a^	1 (3.3)
Fort Frances^a^	1 (3.3)
Hearst^a^	3 (10)
Huntsville	1 (3.3)
Kapuskasing^a^	2 (6.7)
Kenora	2 (6.7)
Manitoulin Island	1 (3.3)
New Liskeard	1 (3.3)
North Bay	3 (10)
Parry Sound	2 (6.7)
Sault Ste. Marie	6 (20)
Sioux Lookout^a^	2 (6.7)
Timmins	4 (13.3)

^a^ *Population < 10,000*

When asked how much classroom based OtoHNS instruction students received during medical school, 90.6% of participants replied ‘very little’. Likewise, an overwhelming majority (88.7%) of students acknowledged receiving ‘very little’ clinical OtoHNS instruction. Student perspectives in OtoHNS instruction are found in Table 2.

**Table 2:** Otolaryngology exposure in undergraduate medical education

Question	Answer choice	n (%)
How much classroom based ENT/Otolaryngology instruction have you received during medical school?	Very little	48 (90.6)
	Adequate	5 (9.4)
	Very adequate	0 (0)
How much clinical ENT/Otolaryngology instruction have you received during medical school?	Very little	47 (88.7)
	Adequate	6 (11.3)
	Very adequate	0 (0)

**Fig. 2:**
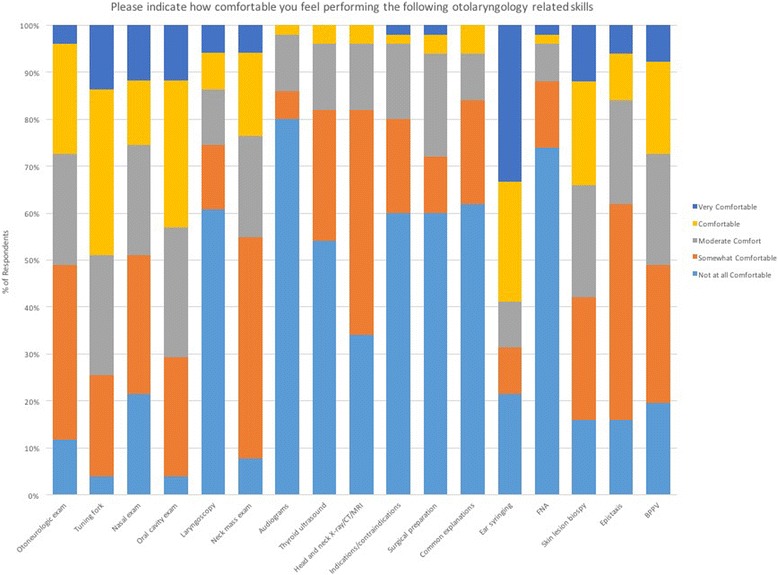
Level of comfort completing otolaryngology procedural skills

**Fig. 3:**
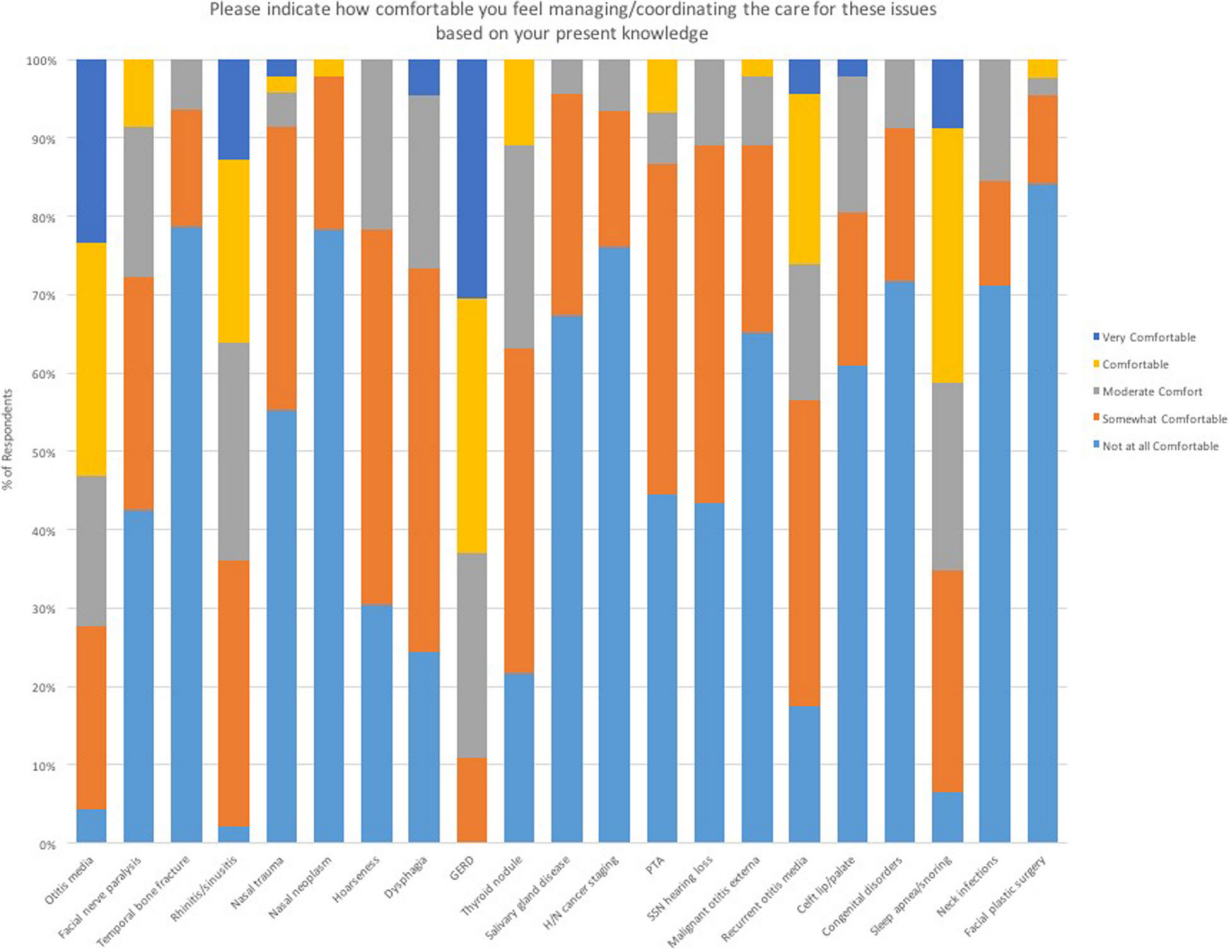
Level of comfort managing common otolaryngology related issues

**Fig. 4:**
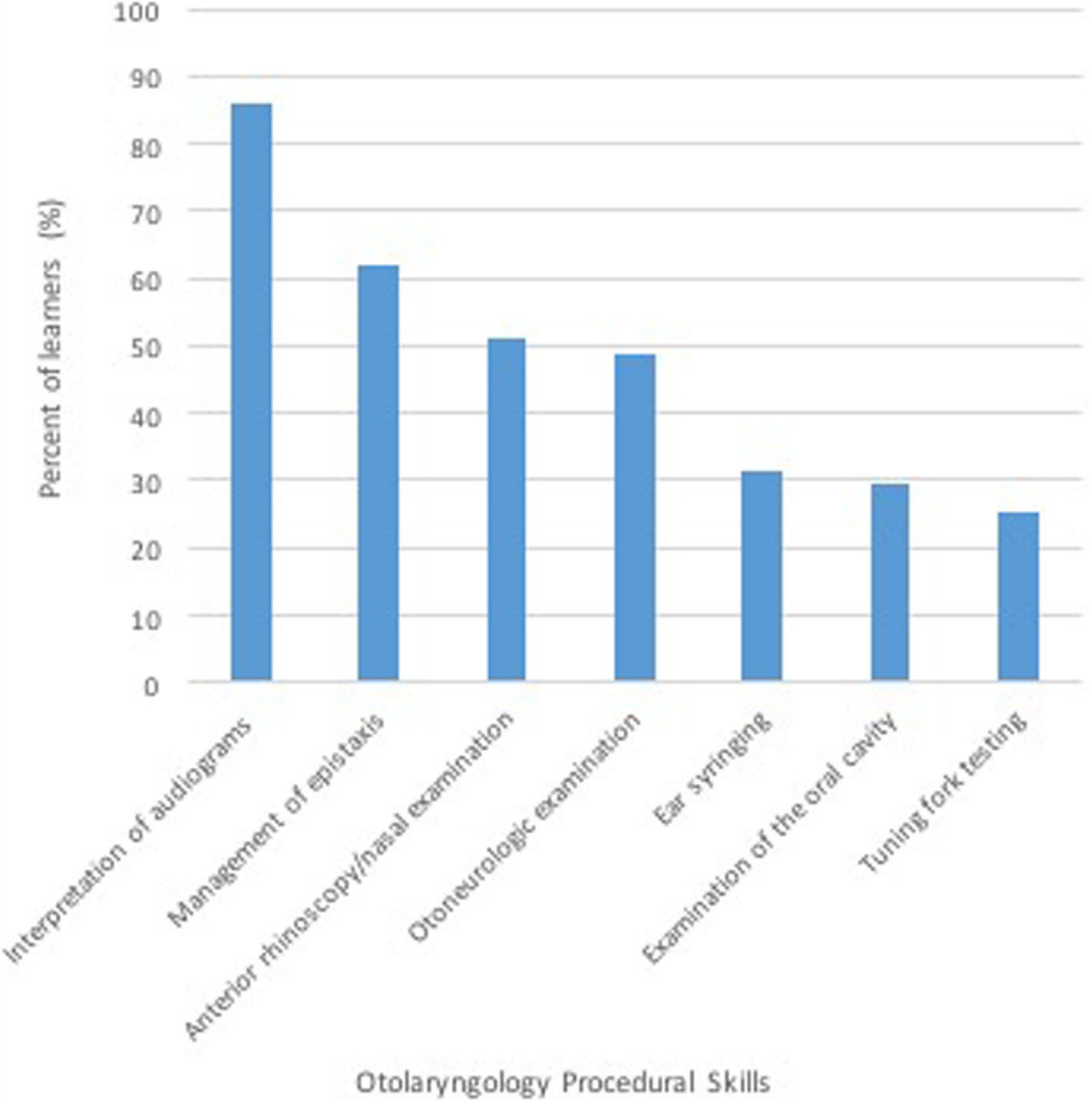
Learners that feel uncomfortable completing common procedural skills

**Fig. 5:**
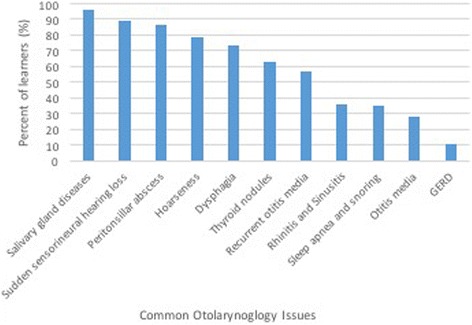
Learners that feel uncomfortable managing common otolaryngology related issues

The second section of the online survey assessed students’ comfort level performing a set of OtoHNS related skills (Fig. 2) and managing/coordinating the care for a set of OtoHNS clinical presentations (Fig. 3). Of the OtoHNS related skills evaluated, the authors identified 7 skills most important for a medical students’ core learning. These skills included interpretation of audiograms and tympanograms, management of epistaxis, anterior rhinoscopy/nasal examination, otoneurologic examination, ear syringing, examination of the oral cavity, and tuning fork testing. These identified skills revealed a wide range of comfort levels. When we group those respondents who selected ‘not at all comfortable’ and ‘somewhat comfortable’ together as a measure of those who were relatively uncomfortable, several trends emerge (Fig. 4). The medical learner participants felt most comfortable with tuning fork testing (25.49% felt not at all or somewhat comfortable) and least comfortable with interpretation of audiograms and tympanograms (86% felt not at all or somewhat comfortable). Of the OtoHNS clinical presentations, the authors identified 11 presentations to be important for a medical students’ core learning. These clinical presentations included salivary gland diseases, sudden sensorineural hearing loss, peritonsillar abscess, hoarseness, dysphagia, thyroid nodules, recurrent otitis media, rhinitis and sinusitis, sleep apnea and snoring, otitis media and gastroesophageal reflux disease. These clinical presentations revealed a wide range of comfort levels. Once again, we grouped those respondents who selected ‘not at all comfortable’ and ‘somewhat comfortable’ together as a measure of those who were relatively uncomfortable (Fig. 5). This revealed that learners felt most comfortable with gastroesophageal reflux disease (10.87% felt not at all or somewhat comfortable) and least comfortable with salivary gland diseases (95.65% felt not at all or somewhat comfortable).

## Discussion

Our study revealed that students are receiving suboptimal exposure to OtoHNS as measured by perceived volume of training and comfort levels. A majority of students surveyed had never observed an otolaryngologist or completed an OtoHNS rotation.

These results are not unique to NOSM. Regardless of clerkship model, OtoHNS remains a challenging competency to acquire. The literature suggests that even those medical students who have completed an OtoHNS rotation reported feeling uncomfortable diagnosing and managing common and emergent conditions [23–25]. This is not surprising considering that the average clerkship time dedicated to OtoHNS in Canada was 4.6 days [7]. Specific gaps have been identified in knowledge surrounding otitis media, tonsillitis, indications for tracheostomy, and airway obstruction as well as specific skill deficits in otoscopy and nasal examination [26]. Considering the importance placed on OtoHNS by primary care practitioners [24, 27], it is imperative that training in OtoHNS be optimized and consistent at the undergraduate medical education level.

With the distributed learning model and LIC, it is essential that OtoHNS curriculum be consistent and equitable for students across teaching sites. There is the need for increased exposure to OtoHNS curriculum without burdening clinical faculty. A review of 17 published studies found students consistently rated clinic-based teaching and small group learning as effective ways to learn OtoHNS [24]. Although case-based and online learning modules are already employed in the distributed learning model at NOSM, further utilization of these learning modalities may help to bridge some of the identified gaps in the OtoHNS curriculum. Simulation has also been shown to be an effective teaching modality that may prove useful in a distributed learning environment where exposure to OtoHNS pathology may vary between clerkship sites. Previous research has echoed the above sentiments by suggesting the need for increased exposure without placing additional burden on clinical faculty [24].

This study assessed OtoHNS exposure and comfort level through the lens of student perspectives. Both exposure to OtoHNS and level of comfort managing common OtoHNS conditions were assessed simultaneously, using a previously validated OtoHNS assessment tool [21]. To our knowledge, this is the first study in the literature to focus on the experience of OtoHNS instruction in a LIC model. However, the authors acknowledge several limitations do exist. The study only captured data from a single time-point at a single Canadian medical school. Additionally, the results may be unique to NOSM as it is the only school with a comprehensive LIC model. Thus, it is conceivable that variation could exist between different medical institutions nationwide and beyond. The anonymity of the survey as well as the moderate response rate may invite concern as to whether the results reflect the perception of all medical students at NOSM. Additionally, there may be a discrepancy in level of comfort and actual knowledge, such that students who feel uncomfortable managing OtoHNS conditions may actually possess the appropriate knowledge.

## Conclusions

Otolaryngology is an important specialty with relevance to family medicine, pediatrics, and general practice. Based on the volume of OtoHNS encounters in primary care practice, there remains an underrepresentation of OtoHNS training at the undergraduate medical education level. Despite geographic distribution of students in the CCC and LIC models, the results of this study were not dissimilar to rotation based clerkship cohorts. Strategies such as standardized objectives and supplemental electronic resources may serve to solidify clinical knowledge with all undergraduate medical learners.

## Additional file

Additional file 1: Appendix 1: Otolaryngology in Undergraduate Medical Education. (DOCX 265 kb)

## Abbreviations

CCC: Comprehensive Community Clerkship
LIC: Longitudinal Integrated Clerkship
NOSM: Northern Ontario School of Medicine
OtoHNS: Otolaryngology Head and Neck Surgery
