# Supplementary material for: Otolaryngology exposure in a longitudinal integrated clerkship setting
Source: J Otolaryngol Head Neck Surg. 2017 Jul 10;46:51. doi: 10.1186/s40463-017-0215-1 (PMC5504570; doi:10.1186/s40463-017-0215-1)
Supplement: Additional file 1: — Appendix 1: Otolaryngology in Undergraduate Medical Education. (DOCX 265 kb) [file 40463_2017_215_MOESM1_ESM.docx]

#
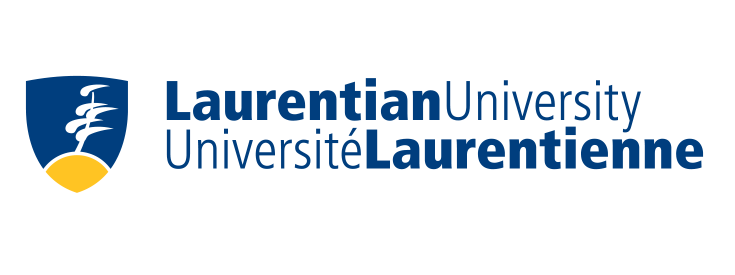

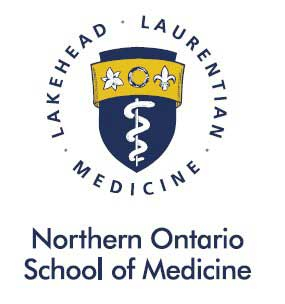


#
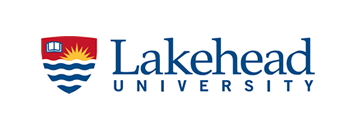


# Otolaryngology in Undergraduate Medical Education

**Questionnaire for Medical Students:**

**1. a) What year of medical school are you presently in?**

2 3 4

**b) If year 3 or above, where did you complete your longitudinal clerkship?**

________________________________________

**2. Have you ever observed an Otolaryngologist?**

Yes No

**3. Have you ever completed an Otolaryngology elective rotation?**

Yes No

**4. If so, please indicate which program you completed an Otolaryngology elective at:**

School Name: __________________________________________

City: __________________________________________

Province: __________________________________________

**5. Which residency are you planning to apply to?**

_______________________________________

**6. Are you considering applying to an Otolaryngology residency program?**

**_______________________________________**

**7. How much classroom based ENT/Otolaryngology instruction have you received during medical school?**

Very little Adequate Very Adequate

**8. How much clinical ENT/Otolaryngology instruction have you received during medical school (i.e. through observerships, clerkship, electives, etc.)?**

Very little Adequate Very Adequate

**9. What component of your Family Medicine exposure can be classified as managing Otolaryngology associated issues (i.e. Otitis Media, Sinusitis, Vertigo, Hoarseness, Thyroid Masses, Head and Neck Masses – including skin cancers, etc.)**

≤ 10% ≤25% ≤ 50% ≤ 75% > 75 %

**10. How comfortable do you feel managing Otolaryngology associated issues? (circle one)**

1 2 3 4 5 6 7 8 9 10

**Very Uncomfortable Very Comfortable**

**11. Using the table below, please indicate how comfortable you feel performing the following Otolaryngology related skills:**

**(1 = Not at all comfortable, 2 = Somewhat comfortable, 3 = Moderate Comfort, 4 = Comfortable, 5 = Very Comfortable)**

| ***Clinical assessment – history and physical*** | 1 | 2 | 3 | 4 | 5 |
| --- | --- | --- | --- | --- | --- |
| Otoneurologic examination |  |  |  |  |  |
| Tuning fork testing |  |  |  |  |  |
| Anterior rhinoscopy/nasal examination |  |  |  |  |  |
| Examination of the oral cavity |  |  |  |  |  |
| Laryngoscopy (indirect) |  |  |  |  |  |
| Examination of a neck mass |  |  |  |  |  |
| ***Investigations*** | | | | | |
| Interpretation of audiograms and tympanograms |  |  |  |  |  |
| Interpretation of the results of a thyroid ultrasound |  |  |  |  |  |
| Interpretation of the results of a head and neck X-ray/CT/MRI |  |  |  |  |  |
| ***Surgical referral, counseling, and care*** | | | | | |
| Indications and contraindications for certain surgical procedures |  |  |  |  |  |
| Preparation of patients for surgery and post-operative care |  |  |  |  |  |
| Explanations of common otolaryngologic surgical procedures  (e.g. myringotomy and tube insertion, tonsillectomy) |  |  |  |  |  |
| ***Procedures*** | | | | | |
| Ear syringing |  |  |  |  |  |
| Fine needle aspiration biopsy |  |  |  |  |  |
| Biopsy of skin lesions for query BCC/SCC |  |  |  |  |  |
| Management of epistaxis |  |  |  |  |  |
| Treatment of benign paroxysmal positional vertigo (BPPV) |  |  |  |  |  |

**12. Using the table below, please indicate how comfortable *you* feel managing/coordinating the care for these issues based on your present knowledge:**

**(1 = Not at all comfortable, 2 = Somewhat comfortable, 3 = Moderate Comfort, 4 = Comfortable, 5 = Very Comfortable)**

| ***Otology*** | 1 | 2 | 3 | 4 | 5 |
| --- | --- | --- | --- | --- | --- |
| Otitis media |  |  |  |  |  |
| Facial nerve paralysis |  |  |  |  |  |
| Temporal bone fractures |  |  |  |  |  |
| ***Rhinosinusology*** | | | | | |
| Rhinitis and sinusitis |  |  |  |  |  |
| Nasal trauma |  |  |  |  |  |
| Nasal neoplasms |  |  |  |  |  |
| ***Laryngology*** | | | | | |
| Hoarseness |  |  |  |  |  |
| Dysphagia |  |  |  |  |  |
| Gastroesophageal reflux disease |  |  |  |  |  |
| ***Head and Neck*** | | | | | |
| Thyroid nodules |  |  |  |  |  |
| Salivary gland diseases |  |  |  |  |  |
| H/N cancer staging and management |  |  |  |  |  |
| ***Otolarynogologic Emergencies*** | | | | | |
| Peritonsillar abscess |  |  |  |  |  |
| Sudden sensorineural hearing loss |  |  |  |  |  |
| Malignant otitis externa |  |  |  |  |  |
| ***Pediatric Otolaryngology*** | | | | | |
| Recurrent otitis media |  |  |  |  |  |
| Cleft lip/palate |  |  |  |  |  |
| Congenital disorders of the neck, mouth, and pharynx |  |  |  |  |  |
| ***Other*** | | | | | |
| Sleep apnea and snoring |  |  |  |  |  |
| Otolaryngologic and deep space neck infections |  |  |  |  |  |
| Facial plastic surgery |  |  |  |  |  |

**13. Please list five common issues/diagnoses you (and or your supervisor) have referred to an Otolaryngologist for:**

- 1. __________________________________________
  2. __________________________________________
  3. __________________________________________
  4. __________________________________________
  5. __________________________________________

This is the end of the questionnaire.

Thank you for taking the time to complete our survey.
